# Mechanistic Explanation of the Weak Carbonic Anhydrase’s Esterase Activity

**DOI:** 10.3390/molecules22061009

**Published:** 2017-06-18

**Authors:** Paolo Piazzetta, Tiziana Marino, Nino Russo

**Affiliations:** Dipartimento di Chimica e Tecnologie Chimiche (CTC), Università della Calabria, 87036 Arcavacata di Rende (CS), Italy; tmarino@unical.it

**Keywords:** carbonic anhydrase, DFT, enzyme promiscuity, enzyme inhibitors

## Abstract

In order to elucidate the elementary mechanism of the promiscuous esterase activity of human carbonic anhydrase (h-CA), we present an accurate theoretical investigation on the hydrolysis of fully-acetylated d-glucose functionalized as sulfamate. This h-CA’s inhibitor is of potential relevance in cancer therapy. The study has been performed within the framework of three-layer ONIOM (QM-high:QM’-medium:MM-low) hybrid approach. The computations revealed that the hydrolysis process is not energetically favored, in agreement with the observed weak carbonic anhydrase’s esterase activity.

## 1. Introduction

Different enzymes, in particular metallo-enzymes, are able to modulate their reactivity, as well as the substrate-selectivity of the system [1]. The tendency to exploit substrate promiscuity, together with advances in protein engineering, allowed extending the reactions promoted by native enzymes to, sometimes, transformations not typical OF the biological world [2,3]. Promiscuous catalysis, proposed in 1921 for explaining the C-C bond formation by pyruvate decarboxylase [4], was considered a dark side of enzyme specificity and, only in 1999, DID this aspect become more popular [5]. Nowadays, enzymatic promiscuity, defined as the possibility of an enzyme to catalyze reactions “in addition to the ones for which they are physiologically specialized or evolve…” is considered an important tool in biotechnology [4,6,7,8].

h-CAs are zinc-containing metallo-enzymes that efficiently catalyze the reversible hydration of carbon dioxide, thereby playing a crucial role in pH regulation [9,10,11,12,13,14,15,16]. In the last years, the interest for h-CA has been considerably grown due to its broad promiscuous activity ranging from substrate to catalytic one [17].

Starting from the early 1960s, when the esterase activity of carbonic anhydrase was observed for the first time [18], several structural, functional and mutational studies showed that the hydratase and esterase activities of CA shared similar mechanisms in the same catalytic pocket [9,19,20,21,22,23,24].

The selective inhibition of h-CA plays a role in the cancer therapy_2_ h-CA exhibits a pronounced activity in CO_2_ hydration (*k_cat_/K_M_*~10^8^ M^−1^ s^−1^) with respect to that of activated esters, such as *p*-nitrophenyl acetate (~10^3^ M^−1^ s^−1^) [19,25,26]. This behavior also accounts for different steric and electronic natures of the implicated substrates. Carbohydrate-based sulfamates having carbohydrate hydroxyl groups in free (-OH) and acylated (-Acyl) forms, showed to be both inhibitors of the CO_2_ hydration and substrates for the esterase activity of h-CA [24]. Since these compounds exhibited good selectivity for cancer-associated h-CA isozymes [27], protein X-ray crystallography and bio-affinity mass spectrometry were used in order to provide structural information about the enzyme-substrate interaction. Both of these biophysical methods indicated that sulfamate compounds initially act as substrates but, following hydrolysis, become inhibitors, thereby competing with the substrate binding mode. These findings are in agreement with the pro-drug strategy largely adopted across medicinal chemistry, where esters are used to mask polar hydroxyl groups [28]. In the fully acetylated D-glucose functionalized as sulfamate, whose hydrolysis reaction by human carbonic anhydrase is the object of the present investigation, the esterification of the acyl group of carbohydrate-based sulfamates is employed to modulate their activity as CA inhibitors [24].

Supported by our previous experience in the investigation on the mechanistic insights of other promiscuous h-CA [29,30,31] reactions, and since the h-CA’s esterase mechanism is still unclear, we have undertaken a theoretical study on the mechanism of h-CA toward a fully-acetylated D-glucose (functionalized as sulfamate) by using a three-layer QM/QM’/MM approach. The obtained results revealed important aspects of the chemical process and, in particular, on the elementary steps of the h-CA hydrolase mechanism. Such information should be important in designing and developing new effective inhibitors.

## 2. Results and Discussion

### 2.1. Enzyme-Substrate Complex

**Figure 1:**
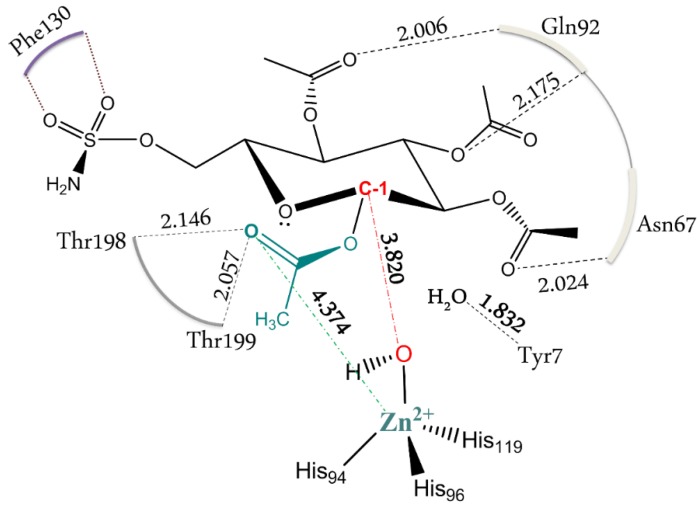
Schematic depiction of the substrate interactions with the surrounding residues in the ES. All of the distances are in Å.

As a first step of the work, we have optimized the enzyme-substrate complex (ES) starting from the previous docking structure [24]. The calculated RMSD referred to the protein backbone of the optimized geometry is 1.51 Å with respect to the initial conformation (see Figure S1). The distance between the oxygen of zinc bound hydroxide and the C-1 carbon of the D-glucose is found to be 3.820 Å and can be considered a reliable distance for the subsequent nucleophilic attack (Figure 1). This topology agrees well with a recent investigation in which it is hypothesized that the substrate first associates with the active site of h-CAII (without coordinating the metal ion) and, in a second step, it coordinates the Zn^2+^ with the –SO_2_NH_2_ moiety (a three states association mechanism) [28]. The pocket of carbonic anhydrase is 15 Å deep and accommodates this substrate, which is much larger than CO_2_, with the participation of residues that usually are not involved in the formation of the molecular complex (Figure 1). It is important to note that the interaction of Phe130 with the –SO_2_NH_2_ tail of the functionalized D-glucose prevents the departure of the substrate itself outside the pocket. The presence of the acetyl groups on the sugar ring increases the steric hindrance preventing movement without involving a concomitant participation of the backbone. Figure 1 shows the electrostatic interactions of the acetyl moieties with the surrounding residues and the role of Tyr7 in retaining a deep water molecule with a hydrogen bond. A hydrophobic interaction of the substrate methylene group with the Thr199 and Leu197 is also present. These structural features are in agreement with a recent investigation which suggests the existence of a pre-binding stage stabilized by a favorable packing of the ligand’s apolar moieties with the hydrophobic portion of h-CA(II) (see Figure 1) [32].

### 2.2. Hydrolysis of the Substrate

**Scheme 1:**
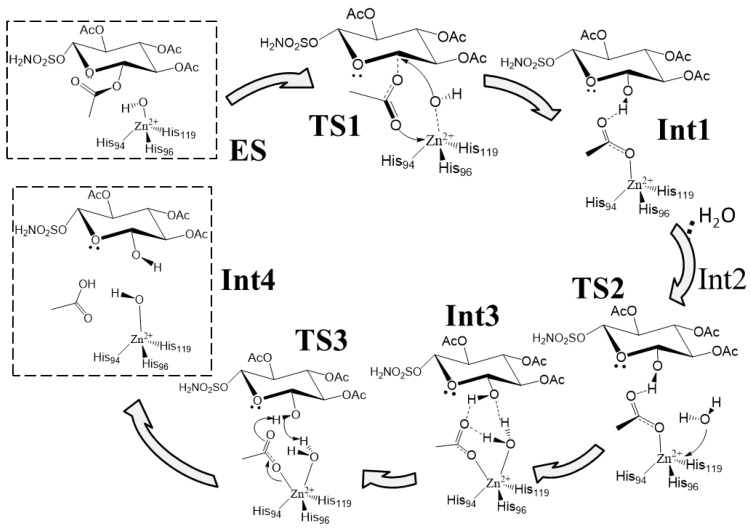
Proposed reaction mechanism.

**Figure 2:**
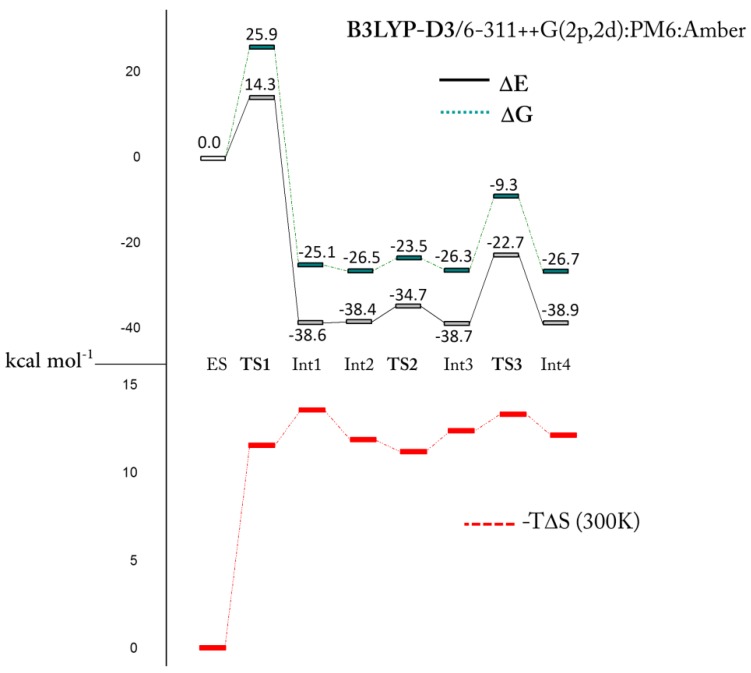
Computed free-energy profile, relative to the ES state, for the investigated esterase mechanism. Gibbs free energies (green), electronic energies (black), and −TΔS (red).

Following some experimental evidence [24], the computed reaction mechanism is shown in Scheme 1, while the related potential energy surface (PES) is reported in Figure 2.

**Figure 3:**
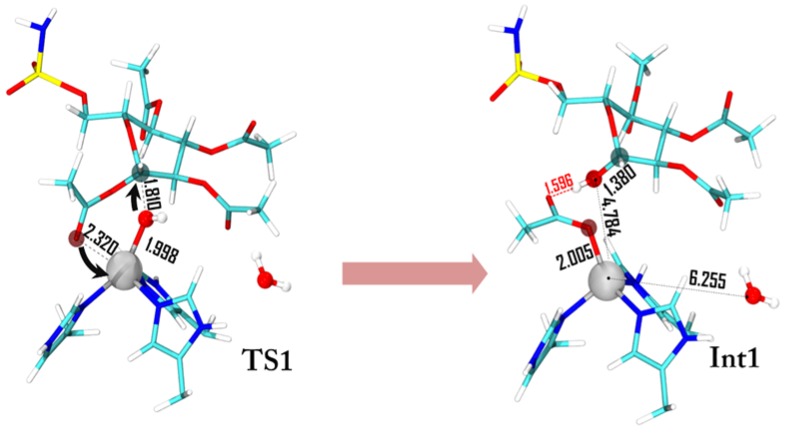
Optimized geometries of the first step of the reaction. For clarity, the low and medium layers are omitted. All of the distances are in Å.

The first step of the mechanism is the nucleophilic attack of the zinc coordinated hydroxyl on the C-1 anomeric carbon. Experimentally, it has been observed that the C-1 suffers an inversion of its configuration during this step. The C-1 acyl group is equatorial in the substrate while the obtained C-1 hydroxyl is axial (95% of the product axial, 5% equatorial) [24]. A two-step mechanism with the formation of an oxacarbenium species due to the coordination of the acyl group on the zinc ion, and a subsequent nucleophilic attack of the OH ion with the formation of the axial product has been proposed. To justify this mechanism, it is necessary to pass through a penta-coordinated zinc complex (20e^−^) after the attack of the acetyl group to the metal center. It is important to note that, usually, zinc prefers to be tetrahedral in the presence of neutral ligands, as histidine residues, and in binding buried sites, as in the case of carbonic anhydrase [33]. Moreover, the transition from an 18e^−^ tetrahedral complex with one negative ligand (OH^−^) to a 20e^−^ penta-coordinated complex with two negative ligands (OH^−^ and CH_3_COO^−^) is highly unlikely. It is known that carbonic anhydrase chemistry allows the formation of a penta-coordinated complex only with the insertion of a neutral ligand (i.e., water) [29,30,31]. Despite these considerations, we tried to simulate this path, but every attempt failed since the penta-coordinated geometry cannot be isolated because during the optimization procedure the hydroxyl ion leaves the coordination sphere. Starting from the evidence that TS1 has to preserve the tetrahedral shape around the metal center, and in order to justify a high percentage of axial product, we suggest a concerted transition state (see Figure 3). The nucleophilic attack of the hydroxyl ion is triggered by the C-1 acyl group that approaches the metal with the carbonyl oxygen promoting the nucleophilic attack of the OH ion towards the anomeric carbon (d_C-1–OH_ = 1.810 Å). The Zn^2+^-OH distance increases from 1.870 Å in the ES to 1.998 Å in the TS1, indicating a lowering of the interaction with the metal.

**Figure 4:**
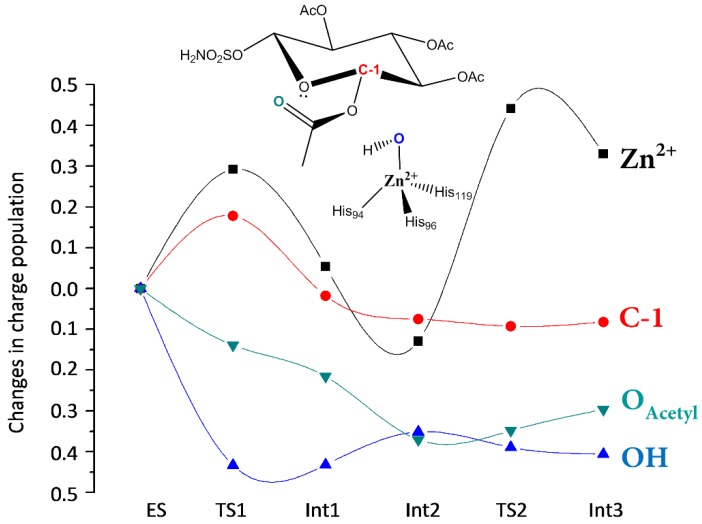
Charge variations for the reactive species occurring along the reaction. A positive value represents an increase in population relative to the ES. Absolute natural charge values for Zn in the path are ES −1.581 |e|, TS1 −1.289 |e|, Int1 −1.527|e|, Int2 −1.710|e|, TS2 −1.140 |e|, Int3 −1.251|e|.

The charge variations (Figure 4) show that the OAcetyl groups smoothly decrease during the interaction with the metal ion, while that of Zn^2+^ increases in the TS1. The charge population of the nucleophilic OH ion decreases consistently with a concomitant increment of that in the anomericC-1 carbon. This behavior indicates that a charge transfer from OAcetyl to Zn^2+^ occurs during the formation of the HO–C-1 bond. The charge variation on Zn^2+^, in going from ES to INT1, is very small since the tetrahedral coordination is restored and the new acetate ligand induces the same charge variation of the detached OH^−^.

Int1 is strongly stabilized and lies at 25.1 kcal·mol^−1^ below ES (see Figure 2). The related geometry is characterized by the zinc coordination with the formed acetate moiety that, in turn, interacts with the sugar ring through a hydrogen bond (Figure 3). The formation of Int1 requires an energetic cost of 25.9 kcal·mol^−1^ [34] (Figure 2) that represents the rate limiting step of the entire chemical process. If we consider that the computed corresponding energy for CO_2_ hydration is about 6 kcal·mol^−1^ [29,34] and the *k_cat_/K_M_* decreases with the increment of the substrate dimension [19,35] our results clearly indicate a weak esterase activity of h-CA. In the TS1 barrier the entropy term (see Figure 2) (−TΔS ≥ 11 kcal·mol^−1^) plays a non-negligible role. The entropic contribution in enzyme processes is often invoked in order to explain their catalytic activity. One example of that is the so-called Jenck’s Circe effect [36] which postulates that enzymes can use part of the substrate-binding free energy to reduce the entropic penalty of the subsequent chemical transformation. This means that part of the binding free energy is used to approach the substrate in the active site, resulting in a loss of translational and rotational entropy of the reactants. Our entropic effect is close to that evidenced by Åqvist et al. [37,38] in some enzymes (about 10 kcal·mol^−1^).

**Figure 5:**
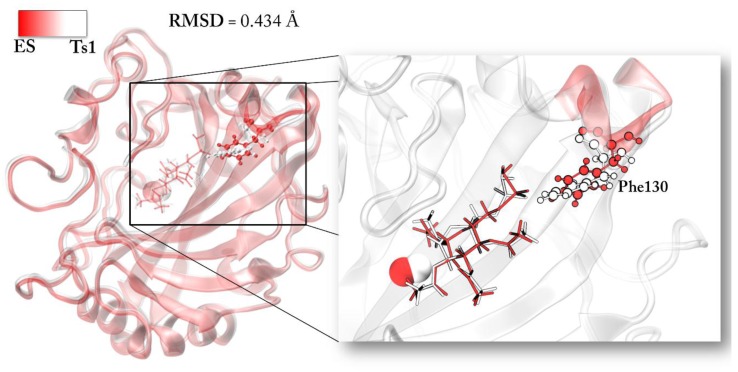
Conformation difference between ES and TS1 species. The calculated RMSD is reported. The hydrogen atoms are omitted.

The superposition of TS1 and ES structures can give insights into this aspect. We find a root mean square deviation (RMSD) of the protein environment related to the TS1 of 0.434 Å with an important deviation in terms of backbone conformation from the ES structure. In particular, the region of the backbone that diverges from the initial conformation includes the Phe130 residue. The steric interaction of this residue with the –SO_2_NH_2_ tail of the functionalized d-glucose is lost during TS1 (see Figure 5). The lack of the Phe130 influence has a direct impact on the distance of the acetylated sugar from the zinc since it is less constrained to be close to the active site.

#### Restoration of the Catalyst

**Figure 6:**
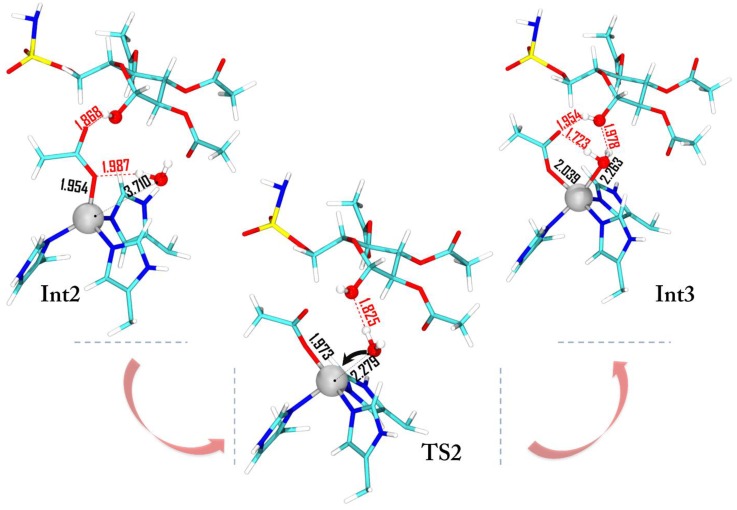
Optimized geometries of Int2, TS2, and Int3. For clarity, the low and medium layers are omitted. All of the distances are in Å.

After the Int1 formation, the reaction proceeds as in the native carbonic anhydrase mechanism: a deep water molecule approaches the zinc ion giving rise to the Int2 (ΔG = −26.5 kcal·mol^−1^) where the water–zinc distance is 3.710 Å (Figure 6). The subsequent intermediate formation Int3 requires an energy of only 3 kcal·mol^−1^ (TS2) and in both cases the penta-coordinated topology is present.

The water insertion on the metal is assisted by a hydrogen bond interaction with the hydroxyl moiety of d-glucose. The charge population variation (Figure 4) indicates that the Zn^2+^ charge increases due to the water molecule coordination while a slight increase is observed for the OAcetyl moiety.

**Figure 7:**
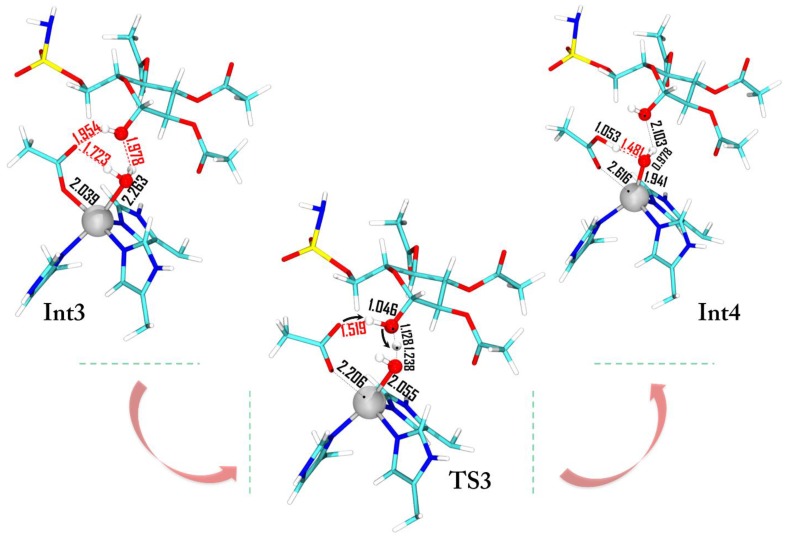
Optimized geometries of the species intercepted in the restoring process. For clarity the low and medium layers are omitted. All the distances are in Å.

Previous investigations on the restoring step of carbonic anhydrase demonstrate that the formation of the penta-coordinated complex represents the key step of the catalytic mechanism of this metallo-enzyme [29,30,31,39]. Many inhibitors (sulfamates or small isoelectronic molecules of carbon dioxide) prevent the water molecule coordination by blocking the enzyme activity. As in the presence of bicarbonate, the acetate needs to be released and the coordinated water molecule must be activated, restoring the nucleophilic species (OH^−^). Several studies suggested that the release of the coordinated product and the formation of the OH^−^ ion could be driven by proton transfers involving the His64 residue through a network of water molecules [40,41]. However, unlike the natural process where the bicarbonate product is directly coordinated to the metal ion and the water is free to diffuse inside the pocket, the sugar is not interacting with the metal (it is still in the cavity), avoiding the water motion and the consequent proton transfer mechanism promoted by the water network. Figure 7 shows the transition state (TS3) with the C-1 hydroxyl moiety that assists the proton shift between the water molecule and the acetate ligand.

The energy of this process lies below ES with a Gibbs activation energy of 17 kcal·mol^−1^ and the final product (Int4) is stabilized by 26.7 kcal·mol^−1^. During the restoring of the hydroxide ion the zinc net charge becomes more negative (Int3 −1.251|e|, TS3 −1.171|e|, Int4 −1.385|e|) assuming almost the same value than that in ES (−1.581|e|). The small difference of Δ|e| = 0.196 is probably due to the influence of the different environment (2.616 Å, see Figure 7).

## 3. Computational Methods

Due to the lack of the X-ray structure for the fully-acetylated d-glucose sulfamate as a substrate of h-CA, we used, as a starting structure, that was kindly given to us by Lopez et al. [24], obtained from a molecular docking on the initial protein X-ray structure PDB ID 3T82.

**Figure 8:**
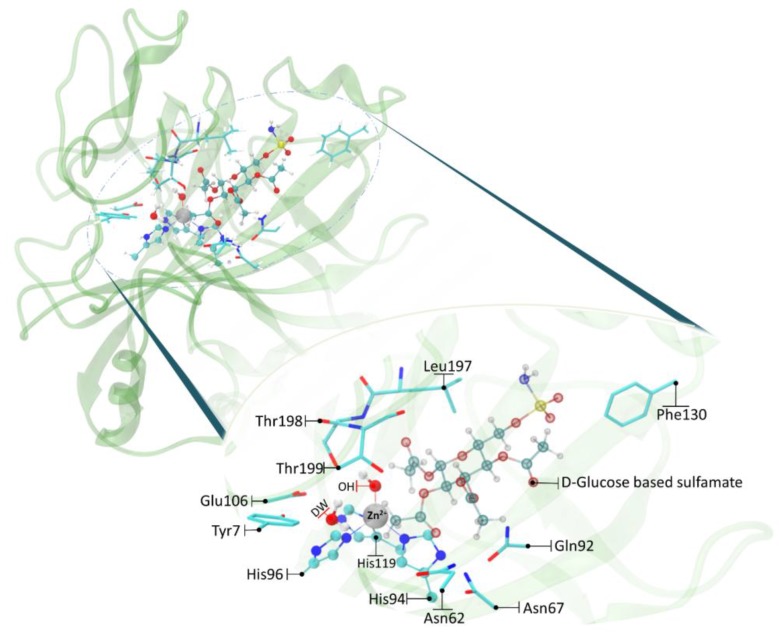
Representation of the h-CA with the fully-acetylated d-glucose functionalized as sulfamate. The high-level layer is illustrated in balls and sticks with the zinc atom as a sphere (88 atoms), the medium-level layer in sticks (106 atoms), and the low-level layer in cartoons (3918 atoms).

In order to take into account the electronic effects deriving by the substrate-enzyme pocket influence, and to achieve a tradeoff between accuracy and computational cost, a three-layer ONIOM [42,43] model in which the active site (88 atoms), the second coordination shell (106 atoms) and the remaining protein portion (3918 atoms) are described at density functional theory (QM), semi-empirical PM6 (QM’), and molecular mechanics (MM) levels, respectively, have been employed [44]. The QM-high layer includes the substrate, the zinc metal ion, and their coordinated histidine residues (94, 96, 119), hydroxyl group, and an explicit free deep water. In the QM’-medium layer, Tyr7, Asn62, Asn67, Gln92, Glu106, Phe130, Leu197, Thr198, and Thr199 have been included. Except for Leu197, Thr198, and Thr199, the remaining ones are truncated as shown in Figure 8. The protein backbone was described at the MM level.

The electronic embedding scheme ONIOM-EE [45], as implemented in Gaussian 09 [46], was used to include the electrostatic interaction between the QM charges and the MM part. The MM charges have been calculated with the charge equilibration model, Qeq, of Rappe and Goddar [47] that allowed to rescale at each computation. B3LYP [48,49], functionally successfully used to describe analogue systems [29,30,50,51], has been employed in conjunction with the 6-31G(d) basis set for C, H, N, S, and O atoms, and the relativistic compact Stuttgart/Dresden effective core potential (SDD) [52] for the zinc metal center. The medium-QM’ level layer has been treated with the semi-empirical PM6 [53] method, previously employed in a recent QM/QM’ study [54], and the MM layer has been described with the Amber force field [55]. All of the intercepted minima and transition states (TS) have been verified by the computation of the vibrational frequencies. Zero point energy (ZPE), thermal energy, and entropy at 300 K have been computed at the B3LYP/6-31G(d):PM6:Amber level of theory. In order to obtain accurate free energies single-point computations with a larger triple-ζ basis set (6-311++G(2d,2p) have been performed on B3LYP/6-31G(d):PM6:Amber-optimized geometries. Non-covalent interactions have been considered by using the D3 parametrization [56] in conjunction with the B3LYP exchange-correlation functional. Furthermore, single point computations on the optimized structures have been performed by also using the M06 [57] functional and the relation is reported in the Supporting Information (Figure S2). In order to evaluate the charge population along the reaction path, the Charge Model 5 (CM5) Hirshfeld charge population analysis [58] has been performed.

## 4. Conclusions

Three-layer ONIOM computations have been performed in order to determine the potential energy surface related to the esterase activity of h-CA. The results show that:

- The rate-limiting step of the process is a concerted event where the C-1 acyl group coordinates the zinc ion with the concomitant nucleophilic attack of the OH ion towards the anomeric carbon.
- The related activation energy is 25.9 kcal·mol^−1^ and can explain the weak esterase activity of carbonic anhydrase.
- The hydrolyzed product shows a consistent energy stabilization with respect to the ES complex, confirming that the fully-acetylated sugar-based sulfamates first bind as a substrate, and after act as an inhibitor.

## Supplementary Materials

The following are available online. Figure S1: Calculated RMSD of the protein backbone of the optimized ES geometry, Figure S2: Gibbs free energies related to the investigated mechanism with M06 functional, Table S1: Electronic energies comparison.
