# Supplementary material for: Mechanistic Explanation of the Weak Carbonic Anhydrase’s Esterase Activity
Source: Molecules. 2017 Jun 18;22(6):1009. doi: 10.3390/molecules22061009 (PMC6152773; doi:10.3390/molecules22061009)
Supplement: Supplementary file 1 [file molecules-22-01009-s001.pdf]

Supporting Information.

Mechanistic explanation of the weak carbonic anhydrase's esterase activity

Paolo Piazzetta[1*], Tiziana Marino[1], Nino Russo[1]

[a]Dipartimento di Chimica e Tecnologie Chimiche (CTC), Università della Calabria, 87036 Arcavacata di Rende (CS) (Italy).

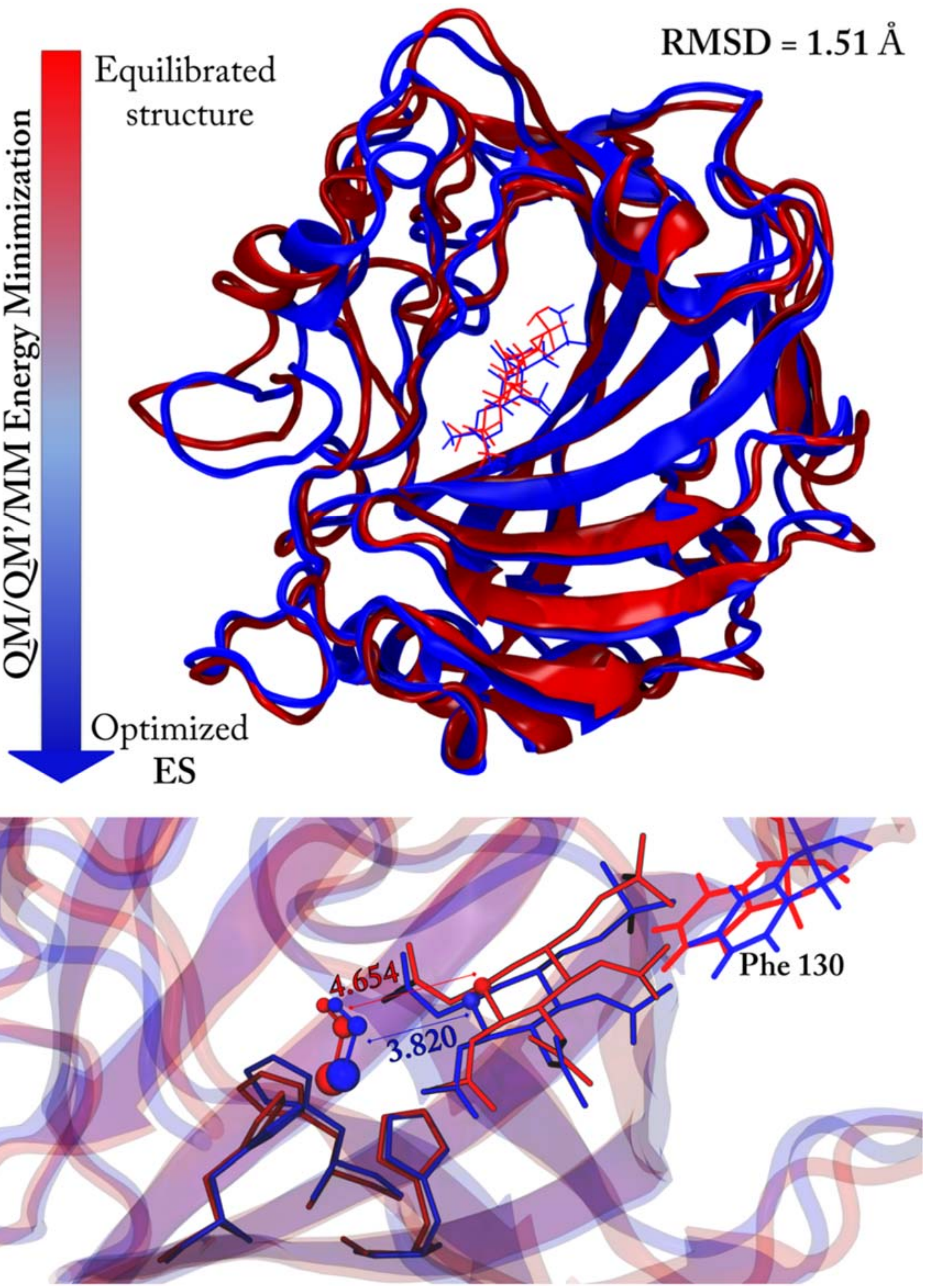

Figure S1 Calculated RMSD of the protein backbone in the optimized geometry respect to the initial conformation. The RED color is associated to the starting geometry while the BLU one is related to the optimized ES.

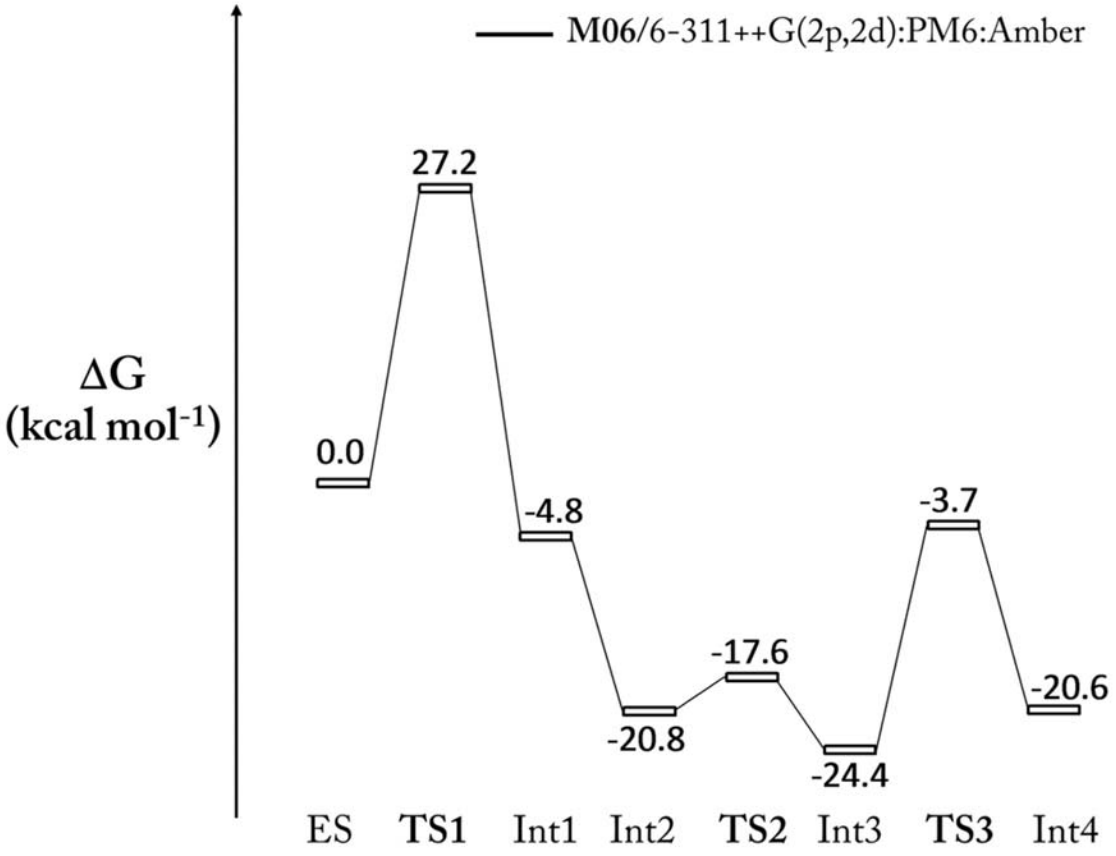

Figure S2 M06 free energies related to the investigated mechanism.

Table S1. Electronic energies comparison at DFT/6-311++G(2d,2p):PM6:AMBER level of theory with B3LYP, B3LYP-D3 and M06 functionals.

	B3LYP ΔE (kcal·mol^{-1})	B3LYP-D3 ΔE (kcal·mol^{-1})	M06 ΔE (kcal·mol^{-1})
**ES**	0.0	0.0	0.0
**TS1**	20.7	14.3	15.6
**Int1**	-38.5	-38.6	-18.3
**Int2**	-36.5	-38.4	-32.7
**TS2**	-29.6	-34.7	-28.9
**Int3**	-39.4	-38.7	-36.8
**TS3**	-16.5	-22.7	-17.1
**Int4**	-36.0	-38.9	-32.8
